# Interpersonal therapy versus antidepressant medication for treatment of postpartum depression and anxiety among women with HIV in Zambia: a randomized feasibility trial

**DOI:** 10.1002/jia2.25959

**Published:** 2022-07-08

**Authors:** M. Bridget Spelke, Ravi Paul, Bryan S. Blette, Samantha Meltzer‐Brody, Crystal E. Schiller, J. M. Ncheka, Margaret P. Kasaro, Joan T. Price, Jeffrey S. A. Stringer, Elizabeth M. Stringer

**Affiliations:** ^1^ Department of Obstetrics and Gynecology University of North Carolina School of Medicine Chapel Hill North Carolina USA; ^2^ University of North Carolina – Global Projects Zambia Lusaka Zambia; ^3^ Department of Psychiatry University of Zambia School of Medicine Lusaka Zambia; ^4^ Department of Biostatistics University of North Carolina Gillings School of Global Public Health Chapel Hill North Carolina USA; ^5^ Department of Psychiatry University of North Carolina School of Medicine Chapel Hill North Carolina USA

**Keywords:** depression, anxiety, postpartum, antidepressive agents, HIV infection, Zambia

## Abstract

**Introduction:**

Postpartum depression (PPD) is a prevalent and debilitating disease that may affect medication adherence and thus maternal health and vertical transmission among women with HIV. We assessed the feasibility of a trial of interpersonal psychotherapy (IPT) versus antidepressant medication (ADM) to treat PPD and/or anxiety among postpartum women with HIV in Lusaka, Zambia.

**Methods:**

Between 29 October 2019 and 8 September 2020, we pre‐screened women 6–8 weeks after delivery with the Edinburgh Postnatal Depression Scale (EPDS) and diagnosed PPD or anxiety with the Mini International Neuropsychiatric Interview. Consenting participants were randomized 1:1 to up to 11 sessions of IPT or daily self‐administered sertraline and followed for 24 weeks. We assessed EPDS score, Clinical Global Impression‐Severity of Illness (CGI‐S) and medication side effects at each visit and measured maternal HIV viral load at baseline and final study visit. Retention, visit adherence, change in EPDS, CGI‐S and log viral load were compared between groups with *t*‐tests and Wilcoxon signed rank tests; we report mean differences, relative risks and 95% confidence intervals. A participant satisfaction survey assessed trial acceptability.

**Results:**

78/80 (98%) participants were retained at the final study visit. In the context of the COVID‐19 pandemic, visit adherence was greater among women allocated to ADM (9.9 visits, SD 2.2) versus IPT (8.9 visits, SD 2.4; *p* = 0.06). EPDS scores decreased from baseline to final visit overall, though mean change was greater in the IPT group (−13.8 points, SD 4.7) compared to the ADM group (−11.4 points, SD 5.5; *p* = 0.04). Both groups showed similar changes in mean log viral load from baseline to final study visit (mean difference −0.43, 95% CI −0.32, 1.18; *p* = 0.48). In the IPT group, viral load decreased significantly from baseline (0.9 log copies/ml, SD 1.7) to final visit (0.2 log copies/ml, SD 0.9; *p* = 0.01).

**Conclusions:**

This pilot study demonstrates that a trial of two forms of PPD treatment is feasible and acceptable among women with HIV in Zambia. IPT and ADM both improved measures of depression severity; however, a full‐scale trial is required to determine whether treatment of PPD and anxiety improves maternal–infant HIV outcomes.

## 1 INTRODUCTION

Depression is a leading global cause of disability [1, 2]. Peripartum depression, defined as an episode of moderate or severe depression beginning during pregnancy or within 4 weeks of delivery [3], is among the most common ailments affecting pregnant and postpartum women [4]. A recent review of the magnitude of postpartum depression (PPD) among women in Africa indicates a prevalence of 7–43% [5]. In Lusaka, Zambia, a study of 229 postpartum women found that 28% met criteria for PPD [6]. Postpartum anxiety disorders, frequently comorbid with postpartum mood disorders, have an estimated prevalence of 10% globally, with higher prevalence in low‐ and middle‐income countries (LMICs) [7].

This staggering disease burden coincides with another, equally staggering one: HIV. HIV prevalence among pregnant women in Lusaka exceeds 20% [8]. Widespread access to combined antiretroviral therapy (ART) has dramatically reduced the rate of perinatal HIV transmission [9]; however, these gains depend upon adherence to prescribed therapies and retention in care [10]. Poor medication adherence is one of the greatest threats to prevention of mother‐to‐child HIV transmission and ART program effectiveness [11, 12, 13, 14]. Unfortunately, the defining symptoms of depression, including sadness, fatigue and withdrawal, may impact a woman's ability to adhere to her medications.

PPD can be treated effectively by medication, psychotherapy or both, with neither approach demonstrating superiority in studies performed in high‐income countries [15]. The most effective treatment for PPD and/or anxiety disorders in women with HIV living in LMICs will vary with access to care, treatment acceptability and adherence. Few studies have evaluated the feasibility of different treatment modalities among women with HIV in sub‐Saharan Africa [16, 17]. We performed a feasibility trial to determine whether a non‐inferiority trial comparing two treatments for PPD among women with HIV would be possible in Lusaka, Zambia. Trial feasibility was assessed by measuring participant retention with the final study visit.

## 2 METHODS

We conducted an un‐masked, un‐blinded, randomized feasibility trial of interpersonal psychotherapy (IPT) versus antidepressant medication (ADM) among postpartum HIV‐positive women with depression and/or anxiety who were receiving ART in Lusaka. Participants were enrolled and followed from 29 October 2019 to 8 September 2020. The trial was registered with clinicaltrials.gov (NCT04094870) and approved by the University of North Carolina Institutional Review Board (17‐3411) and the University of Zambia Research Ethics Committee (011‐11‐18). A study modification was approved in April 2020 to allow for telephonic follow‐up visits aligned with COVID‐19 public health recommendations. All participants completed written informed consent in English, Nyanja or Bemba. Illiterate participants were consented with a literate witness unaffiliated with the study and their consent was documented using a thumbprint.

Participants were recruited from two public postnatal clinics in Lusaka: Chawama First‐level Hospital and Kamwala District Health Clinic. Study staff discussed PPD generally and introduced the study in informational talks. Potential participants were pre‐screened for PPD with the Edinburgh Postnatal Depression scale (EPDS), a 10‐item questionnaire, with four possible responses each assigned 0–3 points, for a score range of 0–30 [18]. Women reporting thoughts of self‐harm were screened for active suicidality. Women with an EPDS score ≥ 6 were invited to provide written informed consent and were assessed for a diagnosis of major depression, anxiety and/or post‐traumatic stress disorder (PTSD) using the Mini‐International Neuropsychiatric Interview (MINI), a brief, structured psychiatric interview [19]. Participants were eligible for randomization if they met the following criteria: (1) ≥ 18 years of age; (2) documented HIV‐1 infection; (3) current ART treatment; (4) 6–8 weeks postpartum from live birth; (5) diagnosis of major depression and/or generalized anxiety on MINI; (6) willingness to provide informed consent; and (7) willingness to adhere to study procedures. We excluded women with: (1) active suicidal thoughts during pre‐screening (defined as thoughts of suicide with a specific plan); (2) known or suspected allergy or contraindication to selective serotonin reuptake inhibitors (SSRIs); (3) history of ADM use within 12 months; or (4) any other condition (social or medical) which, in the study staff's opinion, would make trial participation unsafe or complicate data interpretation. Women experiencing a neonatal death after their most recent delivery were considered to meet this criterion and excluded.

Participants were randomized with equal probability to either ADM or IPT using an electronic system (REDCap) with sealed paper envelopes as backup [20]. A statistician from the UNC Center for AIDS Research Biostatistics Core not associated with the study designed the randomization scheme using random permuted blocks of varying size. All participants were scheduled for 12 visits over 24 weeks, with weekly visits for the first 4 weeks, biweekly visits the next 8 and every 4 weeks thereafter. Study visits were performed by trained research staff in dedicated study space and were not aligned with routine clinical visits. Maternal socio‐demographics, medical and obstetric history were documented at enrolment by reviewing antenatal and HIV‐care cards and by participant report. Four questions adapted from the Humiliation, Afraid, Rape, Kick screening tool assessed for gender‐based violence (GBV) [21]. A follow‐up questionnaire assessed changes in maternal health history 3 months after randomization. Research staff assessed clinical response to treatment at follow‐up visits through repeat EPDS administration and determination of a Clinical Global Impression‐Severity of Illness score (CGI‐S), a 7‐point scale assessing global functioning and mental illness severity [22]. All participants received counselling on the importance of adherence to prescribed medications, including antiretrovirals. We defined study baseline as the enrolment and randomization visit and final visit as the last planned study visit. HIV viral load testing was performed on blood plasma collected at baseline and final study visit with the Cepheid Xpert HIV‐1 Viral Load assay. At the final visit, a satisfaction survey elicited participant agreement with four statements on a 5‐point Likert scale. Adverse events were assessed and graded at each visit using the Division of AIDS Tables for Grading Severity of Adult and Pediatric Adverse Events [23]. Participants requiring additional/crisis treatment were referred to a psychiatrist at the University Teaching Hospital (UTH). An independent data safety monitoring board reviewed cumulative study‐related adverse events every 6 months (NC Translational and Clinical Sciences Institute, NIH UL1TR002489); no additional trial monitoring was performed.

Participants randomized to ADM were instructed to self‐administer a daily oral SSRI (25 mg sertraline) from the day of randomization. Following an evidence‐based treatment algorithm [24], dosage was titrated weekly, in 25 mg increments, until treatment response was achieved based on EPDS and CGI‐S scores. Medication was dispensed in quantities to ensure adequate pills until the next visit; unused medication was documented at subsequent visits. Medication side‐effects were assessed at each visit and dosage was titrated as necessary. At study end, participants were given the option of titrating off sertraline or continuing medication through UTH.

Participants randomized to IPT received up to 11 therapy sessions from trained study nurses. Sessions followed a structured program adapted from the evidence‐based Mental Health Integration Programme, designed for the treatment of depression in patients with HIV in South Africa [25], and included sessions explicitly focused on medication adherence, motherhood, poverty, HIV infection, social isolation and stigma/discrimination as they relate to depression. Research nurses completed an intensive 1‐week, in‐person workshop on the treatment program and received 1 hour of virtual supervision from a licensed psychologist weekly throughout the study.

In Zambia, COVID‐related restrictions on movement were implemented on 17 March 2020. Study procedures were modified to reduce in‐clinic visits. Screening, consent and randomization procedures remained unchanged and required in‐person visits. Participants in the IPT group were given the choice of in‐person or telephonic visits. Participants in the ADM group were assessed for medication tolerance 1 week after randomization and subsequently provided 30 days of medication with monthly medication refills. Follow‐up procedures were completed in‐person or telephonically. All participants were asked to attend the final study visit in person. Exclusively in‐person visits resumed on 15 June 2020.

The primary outcome of this feasibility trial was study retention at final visit. Additional outcomes included: study uptake, adherence with study visits, adherence to treatment, depression/anxiety treatment response, medication toxicity, trial acceptability and change in viral load. We defined study uptake as the percentage of eligible women who agreed to randomization. Adherence with study visits was defined as the mean number of visits completed and the proportion of participants attending ≥ 10 visits. Adherence to treatment was defined differently for each study group. In the ADM group, adherence to treatment was defined as the number of pills taken divided by the expected number of pills taken over the same period. We additionally report the number of ADM participants with adequate adherence, defined as taking >90% of prescribed pills. Participants in the IPT group were considered adherent with a session if they participated in therapy for ≥30 minutes either in person or by telephone. Separately, we report the number of women randomized to the IPT group who completed all sessions. We calculated overall visit and treatment adherence separately for visits scheduled prior to 17 March 2020, which were completed in‐person, and after that date, when in‐person visits were discouraged. Response to treatment was defined as the mean change in EPDS and CGI‐S scores from baseline to final study visit. We also report the number of women with (1) an EPDS score decline of ≥3 points and (2) a CGI‐S score decline of 1 point from baseline to final study visit. We assessed mean change in HIV viral load from baseline to final visit as a preliminary HIV outcome. Finally, we assessed trial acceptability through agreement or disagreement with four statements measured on a 5‐point Likert scale. Responses are reported as strongly agree or agree; neutral; and disagree or strongly disagree.

We calculated mean and standard deviation (SD) for continuous and *n* (%) for categorical baseline characteristics. We compared study attendance at final visit, visit adherence and adverse event frequency between treatment groups. We performed *t*‐tests to compare differences in means between groups and calculated relative risk (RR) and 95% confidence intervals (CI), as appropriate.

Secondary analyses compared mean changes in EPDS, CGI‐S and log viral load from baseline to final visit between study groups using *t*‐tests to calculate mean differences, CIs and *p*‐values. Wilcoxon signed rank tests were used to compare distributions of non‐normal variables. We performed a stratified analysis of change in EPDS by severity of depression at baseline, with mild depression defined as EPDS ≤ 13 and moderate or severe depression defined as EPDS >13.

Within each study group, we performed Fisher's exact tests to compare the proportion of participants at baseline and final visit with: (1) EPDS ≤13; (2) CGI‐S <4; and (3) suppressed viral load. We used univariate linear models to assess for associations between maternal demographic and health characteristics at baseline and treatment adherence. All analyses were performed in R version 4.0.3.

Sample size was based on the minimum desired precision around study retention. We used the normal approximation confidence limit approach to choose a target sample size, with exact binomial CI as a sensitivity analysis. Based on the literature, we anticipated that 70% of randomized participants would be retained at the final visit. Using a 95% CI for the percent of participants retained in the study, a sample size of *n* = 100 ensured a lower confidence limit above 60%. Due to delays in securing sertraline, pandemic related‐disruptions and funding limitations, recruitment ended on 29 April 2020, yielding a final sample size of *n* = 80.

## 3 RESULTS

**Figure 1:**
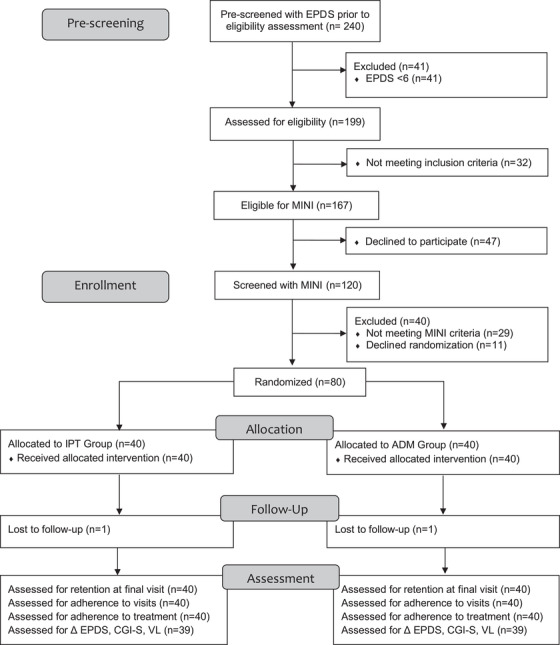
CONSORT flow diagram participant screening and enrolment, 29 October 2019–29 April 2020. Abbreviations: ADM, antidepressant medication; CGI‐S, Clinical Global Impression‐Severity; EPDS, Edinburgh Postnatal Depression Scale; IPT, interpersonal psychotherapy; MINI, Mini International Neuropsychiatric Interview; VL, viral load.

Between October 2019 and April 2020, 240 women completed pre‐screening EPDS. Of those, 199 (83%) had EPDS ≥6 and 167 (70%) met criteria for MINI screening. Of those, 120 (72%) completed MINI evaluation and 91 (76%) met diagnostic criteria. Eighty women were randomized for a study uptake of 88% (95% CI 79–94; Figure 1).

Baseline characteristics were similar between participants randomized to IPT (*n* = 40) and ADM (*n* = 40) (Table 1). Most participants had completed 7–11 years of education, were married/living with a partner and were exclusively breastfeeding their infant. More IPT participants delivered an infant weighing <2500 grams in the antecedent pregnancy (IPT 10/40 [25%] vs. ADM 5/40 [13%]). Fifty‐six participants (72%) reported >1 year since HIV diagnosis. Overall, participants initiated ART a mean 4.1 years (SD 3.2) prior to trial participation. At baseline, 59/80 (74%) had an undetectable viral load (<40 copies/ml). Mean EPDS at baseline was higher among IPT versus ADM participants (14.7 [SD 4.3] vs. 13.1 [SD 3.8]). There was a weak positive correlation between log viral load and EPDS score at baseline (Spearman's correlation coefficient *ρ* = 0.15), though it was not statistically significant (*p* = 0.20). Of the 80 randomized participants, 79 met diagnostic criteria for anxiety (99%), 55 for depression (69%) and 25 for PTSD (31%). Fifty‐four of the 55 participants diagnosed with depression also met criteria for anxiety. More participants in the IPT group than the ADM group reported experiencing GBV (48% vs. 28%; Table 1).

**Table 1:** Baseline characteristics of participants randomized to treatment, 29 October 2019—29 April 2020

Characteristic	Overall (*N* = 80) *n* (%) or mean (SD)	IPT group (*N* = 40) *n* (%) or mean (SD)	ADM group (*N* = 40) *n* (%) or mean (SD)
Maternal age, years	29.7 (5.4)	30.0 (5.3)	29.5 (5.5)
Maternal education			
< 7 years	26 (33%)	12 (30%)	14 (35%)
7–11 years	48 (60%)	27 (68%)	21 (53%)
> = 12 years	6 (8%)	1 (3%)	5 (13%)
Married or living with partner	66 (83%)	35 (88%)	31 (78%)
In poverty	52 (65%)	24 (60%)	28 (70%)
Parity			
1	8 (10%)	3 (8%)	5 (13%)
2–3	41 (51%)	20 (50%)	21 (53%)
> 3	31 (39%)	17 (43%)	14 (35%)
Comorbidities outside pregnancy			
Any prior	5 (6%)	4 (10%)	1 (3%)
Depression	1 (1%)	0	1 (3%)
Antecedent delivery			
Infant birthweight	2900 (490)	2870 (550)	2930 (430)
< 2500 grams	15 (19%)	10 (25%)	5 (13%)
Vaginal delivery	77 (96%)	39 (98%)	38 (95%)
Breastfeeding			
Exclusive	74 (93%)	36 (90%)	38 (95%)
Mixed	6 (7%)	4 (10%)	2 (5%)
Years since HIV diagnosis	4.7 (3.9)	4.3 (3.5)	5.1 (4.2)
≤ 1 year	22 (28%)	10 (25%)	12 (32%)
2–5 years	34 (44%)	21 (53%)	13 (34%)
> 5 years	22 (28%)	9 (23%)	13 (34%)
Missing	2	0	2
Years since ART initiation	4.1 (3.2)	3.9 (3.1)	4.3 (3.3)
≤ 1 year	23 (29%)	11 (28%)	12 (32%)
2–5 years	36 (46%)	21 (53%)	15 (39%)
> 5 years	19 (24%)	8 (20%)	11 (29%)
Missing	2	0	2
Viral load			
< 40 copies/ml	59 (74%)	29 (72%)	30 (75%)
40–1000 copies/ml	8 (10%)	5 (13%)	3 (8%)
> 1000 copies/ml	13 (16%)	6 (15%)	7 (17%)
EPDS at enrolment	13.9 (4.1)	14.7 (4.3)	13.1 (3.8)
≤ 13	41 (51%)	19 (47%)	22 (55%)
> 13	39 (49%)	21 (53%)	18 (45%)
MINI diagnosis at enrolment			
Anxiety	79 (99%)	40 (100%)	39 (98%)
Depression	55 (69%)	29 (73%)	26 (65%)
PTSD	25 (31%)	12 (30%)	13 (33%)
Anxiety and depression	54 (68%)	29 (73%)	25 (63%)
Experienced gender‐based violence	30 (37%)	19 (48%)	11 (28%)
Number of current life stressors	3.2 (2.1)	3.6 (2.3)	2.9 (2.0)

Abbreviations: ADM, antidepressant medication; ART, antiretroviral therapy; EPDS, Edinburgh Postnatal Depression Scale; IPT, interpersonal psychotherapy; MINI, Mini International Neuropsychiatric Interview; PTSD, post‐traumatic stress disorder.

Study retention at final visit was 78/80 (98%, CI 85–99%) with 39/40 participants (98%) in each group completing final visit (Figure 1 and Table 2). Participants attended a mean of 9.4 of 12 visits (SD 2.4) and 45 participants (56%) attended ≥10 visits (Table 2). Visit adherence was greater among ADM participants, although this difference was not significant (Table 2). A stratified analysis of visit adherence indicated that the proportion of completed study visits in both groups decreased after COVID‐19 restrictions went into effect (0.89 [SD 0.18] vs. 0.74 [SD 0.24]; Table 2 and Figure S1). Prior to COVID‐19 restrictions, adherence to treatment was similar between both groups (IPT 0.85 [SD 0.22] vs. ADM 0.82 [SD 0.10]). Adherence with IPT sessions decreased after telephone visits were introduced (from 0.85 [SD 0.22] to 0.71 [SD 0.26]; Table 2). In the IPT group, 5/40 (13%) participants attended all 11 therapy sessions. In the ADM group, 14/40 (35%) participants adhered with > 90% of prescribed pills. Three participants in the ADM group changed their ART medications during the study period and none in the IPT group. Low infant birthweight was significantly negatively associated with adherence to sertraline (*p* = 0.01). No other baseline clinical or demographic characteristics were associated with treatment adherence in either group (Table S1).

**Table 2:** Trial feasibility and acceptability outcomes: overall and by study group

Outcome	Overall (*N* = 80) *n* (%) or mean (SD)	IPT group (*N* = 40) *n* (%) or mean (SD)	ADM group (*N* = 40) *n* (%) or mean (SD)	Mean difference or relative risk (95% CI)	*p*‐value
Attended final study visit	78 (98%)	39 (98%)	39 (98%)	1.00 (0.06, 15.4)	1
Number visits attended	9.4 (2.4)	8.9 (2.4)	9.9 (2.2)	1.00 (−0.04, 2.04)	0.06
< 6	7 (9%)	5 (12%)	2 (5%)	Referent	
6–9	28 (35%)	17 (43%)	11 (28%)		0.05
> = 10	45 (56%)	18 (45%)	27 (67%)	1.69 (1.00, 2.87)	
Proportion of visits attended	0.78 (0.20)	0.74 (0.21)	0.82 (0.18)	0.08 (0.00, 0.17)	0.06
Before 17 March 2020	0.89 (0.18)	0.85 (0.22)	0.94 (0.13)	0.09 (0.00, 0.18)	0.06
After 17 March 2020	0.74 (0.24)	0.71 (0.26)	0.77 (0.22)	0.06 (−0.05, 0.16)	0.31
Adherence to treatment^a^	0.81 (0.16)	0.74 (0.21)	0.85 (0.11)	–	–
Before 17 March 2020	0.83 (0.17)	0.85 (0.22)	0.82 (0.10)	–	–
After 17 March 2020	0.79 (0.22)	0.71 (0.26)	0.88 (0.10)	–	–
Adverse events					
Adverse drug reaction, Grade 1	7 (9%)	0	7 (18%)	15.0 (0.89, 254)	0.06
Severe depression, Grade 2	1 (1%)	0	1 (3%)	3.00 (0.13, 71.5)	0.50
Suicidal ideation, Grade 2	3 (4%)	2 (5%)	1 (3%)	0.50 (0.05, 5.30)	0.56
Referred to UTH psychiatry	8 (10%)	5 (12%)	3 (8%)	0.60 (0.15, 2.34)	0.46
Received non‐study ADM	–	4 (10%)	–	–	–
Opted ongoing treatment at study end	–	–	22 (55%)	–	–
Participant Satisfaction Survey (*N* = 78)					
Happy to have been in study					
Agree or strongly agree	78 (100%)	39 (100%)	39 (100%)	1.00 (0.02, 49.2)	
Neutral	0	0	0	Referent	1
Disagree or strongly disagree	0	0	0		
Feels better now					
Agree or strongly agree	78 (100%)	39 (100%)	39 (100%)	1.00 (0.02, 49.2)	
Neutral	0	0	0	Referent	1
Disagree or strongly disagree	0	0	0		
Did not mind intervention					
Agree or strongly agree	74 (95%)	37 (95%)	37 (95%)	1.00 (0.15, 6.75)	
Neutral	0	0	0	Referent	1
Disagree or strongly disagree	4 (5%)	2 (5%)	2 (5%)		
Would rather have been in other arm					
Agree or strongly agree	10 (13%)	4 (10%)	6 (15%)	1.50 (0.46, 4.90)	
Neutral	26 (33%)	16 (41%)	10 (26%)	Referent	0.50
Disagree or strongly disagree	42 (54%)	19 (49%)	23 (59%)		

Abbreviations: ADM, antidepressant medication; EPDS, Edinburgh Postnatal Depression Scale; IPT, interpersonal psychotherapy; UTH, University Teaching Hospital.

^a^ Adherence to IPT defined as proportion of IPT sessions attended for ≥30 minutes. Adherence to ADM defined as proportion of pills prescribed less pills remaining over expected number of pills taken between two visits.

**Figure 2:**
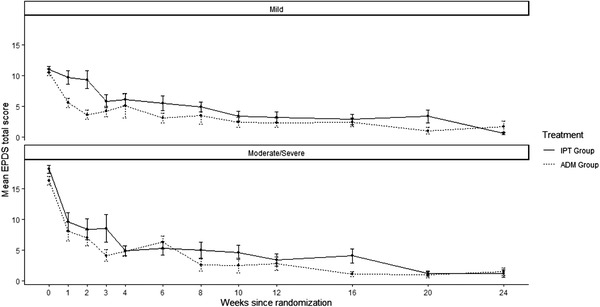
Mean Edinburgh Postnatal Depression Scale (EPDS) across time by treatment group and depression severity at baseline. Abbreviations: ADM, antidepressant medication; EPDS, Edinburgh Postnatal Depression Scale; IPT, interpersonal psychotherapy. Mild depression defined as EPDS score ≤ 13; moderate/severe depression defined as EPDS score > 13. Error bars represent standard error around the mean.

From study baseline to final visit, 37/39 ADM participants and 39/39 IPT participants had a decline in EPDS of ≥ 3 points. Mean EPDS decreased from baseline to final visit in both treatment groups (Table 3 and Figure 2). There was a greater decrease in mean EPDS among IPT participants (−13.8 points, SD 4.7) compared to ADM participants (−11.4 points, SD 5.5, *p* = 0.04). Changes in CGI‐S scores revealed similar results: 39/39 ADM participants and 38/39 IPT participants had a CGI‐S score decline of ≥1 point. Mean CGI‐S scores decreased in both groups across the duration of the study, with greater reduction in mean CGI‐S scores among IPT participants (−3.4, SD 1.0) compared to ADM participants (−2.9, SD 1.3; *p* = 0.06) (Table 3).

**Table 3:** Change in depression scores and HIV viral load between study groups, baseline to final study visit

Outcome	IPT group (*N* = 39) mean (SD)	ADM group (*N* = 39) mean (SD)	Mean difference (95% CI)	*p*‐value^b^
Δ EPDS	−13.8 (4.7)	−11.4 (5.5)	2.44 (0.13, 4.74)	0.04
Δ CGI‐S	−3.4 (1.0)	−2.9 (1.3)	0.49 (0.03, 1.00)	0.06
Δ Log viral load^a^	−0.7 (1.6)	−0.3 (1.7)	−0.43 (−0.32, 1.18)	0.48

Abbreviations: ADM, antidepressant medication; CGI‐S, Clinical Global Impression‐Severity; EPDS, Edinburgh Postnatal Depression Scale; IPT, interpersonal psychotherapy.

^a^ Calculated as the log base 10 VL, in copies/ml. Adding an integer of 1 to each observed viral load allowed log‐transformation of undetectable (zero) values.

^b^ Calculated by *t*‐tests (EPDS and CGI‐S) and Wilcoxon signed rank test (log VL).

**Figure 3:**
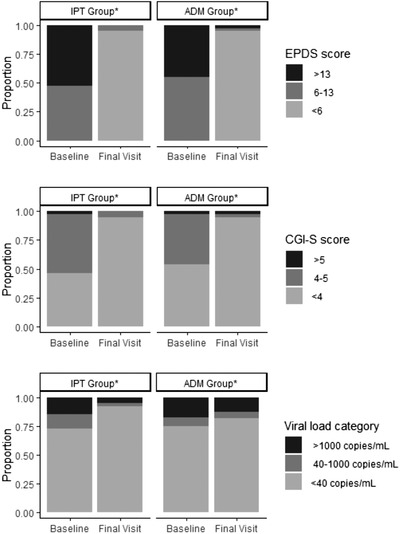
Depression scores and viral load at baseline and final visit by study group. Abbreviations: ADM, antidepressant medication; CGI‐S, Clinical Global Impression‐Severity; EPDS, Edinburgh Postnatal Depression Scale; IPT, interpersonal psychotherapy. ^*^Indicates *p* <0.001.

The proportion of participants with viral suppression significantly increased from baseline to final visit in both groups (Figure 3). At the final visit, 36 participants (92%) in the IPT group and 32 participants (82%) in the ADM group were virally suppressed (Figure 3); this difference was not statistically significant (*p* = 0.19). Mean log viral load decreased among IPT participants (baseline 0.9 [SD 1.7], final visit 0.2 [0.9], *p* = 0.01). Though change in mean log viral load was not significant within the ADM group (baseline 1.0 [SD 1.8], final visit 0.7 [SD 1.6], *p* = 0.33), mean changes in log viral load were similar in both groups (mean difference −0.43 [CI −0.32, 1.18], *p* = 0.48; Table 3).

Adverse events were documented in eight (20%) participants randomized to ADM; seven participants had mild adverse drug reactions, and one participant was diagnosed with severe depression (EPDS >20) and suicidal ideation (Table 2). No participants discontinued their medications due to adverse drug reactions. In the IPT group, two participants reported suicidal ideation and required further care. Five participants in the IPT group were referred to UTH for additional treatment, four of whom were prescribed non‐study ADM by a UTH psychiatrist.

All 78 participants who completed the satisfaction survey reported being “happy to have been in the study” and agreed or strongly agreed with the statement that they “feel better now” as compared to study start (Table 2). In each group, 37/39 (95%) reported that they did not mind the assigned study intervention, while 2/39 (5%) reported that they did. In the IPT group, 4/39 (10%) indicated that they would have rather been in the ADM group, while 6/39 (15%) ADM participants indicated a preference for the IPT group (Table 2).

## 4 DISCUSSION

In this pilot study assessing the feasibility of two different therapies for PPD or anxiety in women living with HIV, we identified and recruited women with these diagnoses into a randomized trial and followed them over a 6‐month period. The study exceeded its goal for participant retention and achieved high study uptake and treatment adherence, despite COVID‐19 disruptions. Participants reported high acceptability of the trial and for both treatment modalities. These findings indicate that a phase III efficacy trial for the treatment of postpartum mental health disorders among women with HIV would be feasible and acceptable in Lusaka, Zambia.

The prevalence of PPD in LMICs is high, at 14–23%, with similar prevalence reported from studies in Africa [6, 26, 27]. While many published reports from sub‐Saharan Africa estimate the prevalence of perinatal mental health disorders, few evaluate treatment strategies. A systematic review of interventional studies for the treatment of PPD identified 18 studies from LMICs, and only one that included medication management [17]. Our study is novel in that it adds to the few published treatment trials for PPD and anxiety in sub‐Saharan Africa and assesses the feasibility of different treatment modalities for both mental health and HIV outcomes.

Few studies have evaluated the effects of medication treatment for depression on ART adherence and HIV outcomes. In the United States, patients taking SSRIs had improved ART adherence and viral load suppression compared to patients who were not depressed [28, 29]. A randomized trial among people living with HIV and depression in South Africa showed that people receiving cognitive behavioural therapy delivered by nurses were more than twice as likely to be virologically suppressed at 12 months compared to people receiving standard care [30]. While our pilot trial was not powered to assess treatment efficacy for mental health and HIV outcomes, both groups showed significant declines in EPDS and CGI‐S scores from baseline to final visit indicating improvement in symptoms following either treatment modality. HIV viral load significantly decreased from baseline to final visit in the IPT group. Further studies are needed to evaluate the impact of different treatments for postpartum mental health disorders on HIV outcomes.

Each treatment modality for PPD has potential strengths and weaknesses, particularly in countries with few mental health providers, limited access to care and high comorbidity with anxiety and stress disorders. IPT has been shown to be effective for treating depression among HIV‐positive women experiencing GBV in Kenya [31] and group IPT decreased depression scores among villagers in Uganda [32]. Consistent with studies in South Africa, we found that mid‐level healthcare providers could provide structured IPT using a manual which was effective in treating depression and anxiety [33]. Medication‐based treatment for PPD is widely recognized as safe and no participants discontinued sertraline during the trial [34, 35]. Our trial reports greater visit adherence in the ADM group; however, this finding should be interpreted in the context of the COVID‐19 pandemic when national policies limited in‐person visits. Notably, a randomized trial comparing group therapy to medication treatment among women with PPD in Zimbabwe found a greater decrease in mean EPDS among women receiving group therapy and no impact on ART adherence [36]. A strength of our study was use of the MINI to diagnose women with depression, anxiety and PTSD. Over 30% of participants were diagnosed with PTSD. Future work should aim to identify and treat postpartum anxiety and stress disorders in Zambia and incorporate interventions that can treat both depressive and trauma disorders.

This study has several limitations. First, the target sample size was not achieved due to delays in securing sertraline and pandemic‐related disruptions. The initial sample size calculation assumed loss to follow‐up of 30%; however, only 2/80 participants (2.5%) were lost to follow‐up, indicating that our final sample size was adequate. Second, the COVID‐19 pandemic required that we adjust study procedures mid‐trial by introducing telephone counselling and dispensing greater quantities of medication, which may have affected trial outcomes. Third, trained research staff provided mental health interventions and assessed clinical improvement. In a full‐scale trial, engaging research staff not involved in treatment to assess clinical outcomes may reduce outcome bias. Despite randomization, a greater proportion of IPT participants than ADM participants reported experiencing GBV, which may have affected treatment response. Finally, participants were randomized to two treatment interventions with no control group. A longitudinal study of perinatal depression in South Africa suggested moderate decreases in the prevalence of depression over time without intervention (17% at 10 weeks to 15% at 6 months) [37]. Though screening and treatment for PPD is not routinely offered in Zambia, we felt that it would have been unethical to identify women with mental illness and not provide treatment.

## 5 CONCLUSIONS

Treating women with HIV and PPD has the increased benefit of potentially improving adherence to antiretrovirals and long‐term maternal and infant HIV outcomes. This pilot study demonstrates that postpartum women with HIV will agree to depression screening and participate in a 6‐month treatment trial. Importantly, women who participated in the study felt that treatment was beneficial and trial participation was acceptable. Future large‐scale trials are needed to assess the efficacy of treating common mental health conditions in the postpartum period on maternal and infant HIV outcomes.

## COMPETING INTERESTS

The authors declare that they have no competing interests.

## AUTHORS’ CONTRIBUTIONS

EMS, RP, SMB, CES and JSAS contributed to the conception and design of the study. EMS, MPK, MBS and JMN contributed to the acquisition of the data. MBS, EMS, BSB, CES and MPK contributed to data analysis and all authors (MBS, RP, BSB, SMB, CES, JMN, MPK, JTP, JSAS and EMS) contributed to interpretation of the data. MBS and EMS drafted the manuscript. All authors critically revised the manuscript and provided final approval for publication.

## FUNDING

This study was supported by a grant from National Institute of Mental Health (PI: Stringer, E.; NIMH 1R21MH115806). We acknowledge the editorial assistance of the NC Translational and Clinical Sciences (NC TraCS) Institute, which is supported by the National Center for Advancing Translational Sciences (NCATS), National Institutes of Health, through Grant Award Number UL1TR002489. Trainee support was provided through the US National Institutes of Health for MBS (T32 HD075731; D43 TW009340) and JTP (K01 TW010857).

## Supporting information


**Figure S1**. Proportion of Study Visits Attended by Date, All participants (n=80).
**Table S1**. Unadjusted Association between Baseline Characteristics and Adherence to Treatment by Study Group.
