# Supplementary material for: Interpersonal therapy versus antidepressant medication for treatment of postpartum depression and anxiety among women with HIV in Zambia: a randomized feasibility trial
Source: J Int AIDS Soc. 2022 Jul 8;25(7):e25959. doi: 10.1002/jia2.25959 (PMC9270230; doi:10.1002/jia2.25959)
Supplement: Supplementary file 1 — Figure S1. Proportion of Study Visits Attended by Date, All participants (n=80). Table S1. Unadjusted Association between Baseline Characteristics and Adherence to Treatment by Study Group. [file JIA2-25-e25959-s001.docx]

**Supplemental Table 1. Unadjusted Association between Baseline Characteristics and Adherence to Treatment by Study Group**

|  | **IPT Group** | | |  | **ADM Group** | | |
| --- | --- | --- | --- | --- | --- | --- | --- |
| **Characteristic** | coefficient | 95% CI | P-value^§^ |  | coefficient | 95% CI | P-value^§^ |
| Maternal age, years | 0.91 | (-0.33, 2.15) | 0.15 |  | -0.31 | (-0.79, 0.16) | 0.19 |
| Married or living with partner | -6.91 | (-26.9, 13.1) | 0.49 |  | -1.24 | (-7.57, 5.08) | 0.69 |
| In poverty | 3.47 | (-10.1, 17.0) | 0.61 |  | 1.49 | (-4.28, 7.25) | 0.60 |
| Parity | 8.69 | (-1.75, 19.1) | 0.10 |  | -1.93 | (-6.01, 2.14) | 0.34 |
| Infant birthweight < 2500g | -3.06 | (-18.4, 12.3) | 0.69 |  | -9.64 | (-17.0, -2.32) | 0.01 |
| Vaginal delivery, n (%) | -9.62 | (-52.1, 32.9) | 0.65 |  | 7.66 | (-4.18, 19.5) | 0.20 |
| Years since HIV diagnosis | 0.77 | (-1.11, 2.66) | 0.41 |  | 0.25 | (-0.39, 0.89) | 0.43 |
| Number of current life stressors | 2.40 | (-0.46, 5.25) | 0.10 |  | 0.75 | (-0.59, 2.08) | 0.27 |
| EPDS at enrollment | -0.13 | (-1.69, 1.43) | 0.87 |  | 0.40 | (-0.29, 1.08) | 0.25 |
| Experienced GBV | 7.00 | (-6.13, 20.1) | 0.29 |  | -0.92 | (-6.84, 5.01) | 0.76 |

IPT, interpersonal psychotherapy; ADM, antidepressant medication; EPDS, Edinburgh Postnatal Depression Scale; GBV, gender based violence.

^§^Calcuated by univariate linear models


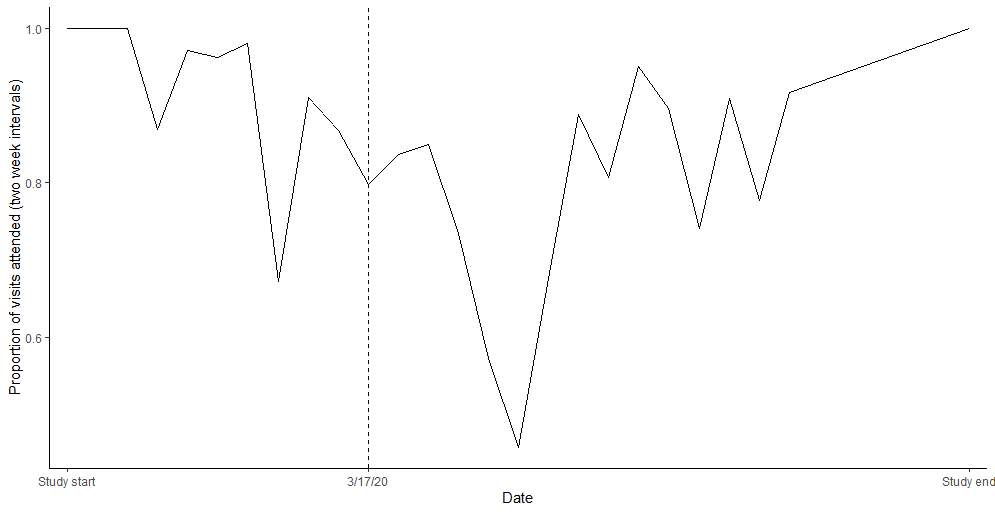
 **Supplemental Figure 1. Proportion of Study Visits Attended by Date, All participants (n=80)**
